# Development and validation of multiple machine learning models for identifying factors associated with walking ability in ischemic stroke patients: a single-center retrospective study with SHAP approach

**DOI:** 10.3389/fneur.2026.1854073

**Published:** 2026-09-01

**Authors:** Jianying Qiu, Xiaoguang Yang, Meng Guo, Wei Guo, Xinyi Guo, Xiping Jia, Qianqian Sun

**Affiliations:** 1 School of Electrical Engineering, Guangdong Songshan Polytechnic College, Shaoguan, Guangdong, China; 2 Department of Rehabilitation Medicine, Xiangyang Central Hospital, Affiliated Hospital of Hubei University of Arts and Science, Xiangyang, Hubei, China; 3 Department of Medical Records and Statistics, Xiangyang Central Hospital, Affiliated Hospital of Hubei University of Arts and Science, Xiangyang, Hubei, China; 4 School of Computer Science, Guangdong Polytechnic Normal University, Guangzhou, China; 5 Rehabilitation Medicine and Rehabilitation Engineering Technology Xiangyang Key Laboratory, Xiangyang, Hubei, China

**Keywords:** influencing factor, ischemic stroke, machine learning, neutrophil-to-lymphocyte ratio, walking ability

## Abstract

**Objectives:**

The aim of this study was to develop and validate machine learning (ML) models that integrate inflammatory biomarkers with clinical indicators to identify factors associated with walking ability in patients with ischemic stroke (IS). The major research question was which ML model achieves optimal discriminative performance for gait impairment in IS patients, and which factors are the key factors of walking ability in this population.

**Methods:**

This retrospective cohort study enrolled 1,650 patients diagnosed with ischemic stroke. The participants were randomly allocated to a training set (70%) and a validation set (30%). Data on clinical, laboratory, and imaging variables were collected. Feature selection was performed using the Least Absolute Shrinkage and Selection Operator (LASSO) regression, the Boruta algorithm, and logistic regression. Six machine learning models were developed. SHapley Additive exPlanations (SHAP) analysis was applied to the best-performing model.

**Results:**

Five significant factors were screened: the neutrophil-to-lymphocyte ratio (NLR), age, gender, occipital lobe and frontal lobe lesions. Random forest (RF) achieved optimal performance with an AUC of 0.868 in the training set and 0.681 in the test set. SHAP analysis prioritized NLR as the top contributor.

**Conclusions:**

The ML models show preliminary promise as screening tools for early risk stratification for gait impairment in IS patients.

## 1 Introduction

Globally, stroke remains the second leading cause of mortality and the primary cause of disability among adults (1, 2). In 2021, the global prevalence of stroke reached 93.8 million cases, with ischemic stroke (IS) accounting for 65.3% (3). Despite advancements in medical technology that have contributed to reduced stroke mortality, over 60% of survivors develop varying degrees of hemiplegia (4). Gait impairment emerges as one of the most prevalent and disabling motor deficits after stroke (4, 5). Gait impairment significantly limits activities of daily living (including transfers and stair climbing), elevates the risk of falls, and predisposes individuals to secondary complications such as joint contractures and muscle atrophy (6–8). These not only diminish quality of life but also impose considerable caregiver burden and healthcare economic costs, with the global economic burden of stroke projected to exceed 1.8 trillion by 2050 (3). Given these challenges, the restoration of walking ability represents a central objective in stroke rehabilitation. A comprehensive assessment of factors influencing walking dysfunction is essential for formulating individualized therapeutic strategies and optimizing the allocation of rehabilitation resources.

Post-ischemic inflammation is a critical pathophysiological process contributing to secondary neural injury and functional impairment after cerebral infarction (9, 10). Extensive clinical and experimental evidence indicates that acute inflammatory responses exacerbate blood-brain barrier disruption, vasogenic edema, and neuronal damage, ultimately influencing neurological outcomes (11). In parallel, systemic inflammation promotes atherosclerosis progression and thrombotic events, further amplifying ischemic injury (12). Recently, complete blood count (CBC)-derived inflammatory indices, such as the neutrophil-to-lymphocyte ratio (NLR), systemic immune-inflammation index (SII), and systemic inflammation response index (SIRI), have emerged as accessible and cost-effective biomarkers for evaluating functional prognosis in IS patients. However, existing research has primarily focused on global stroke risk assessment rather than domain-specific deficits. The relationship between inflammatory biomarkers and post-stroke walking ability remains poorly defined.

This study aims to develop and validate a simple and interpretable risk stratification model for gait ability in patients with ischemic stroke (IS) using multimodal data, including stroke location, inflammatory markers, and clinical characteristics, thereby enabling early and personalized risk stratification. Strongly associated factor variables were initially identified through a combination of Least Absolute Shrinkage and Selection Operator (LASSO) regression, Boruta algorithm, and logistic regression analysis. Machine learning (ML) models were subsequently employed to determine the optimal risk assessment model and key factors for rapidly estimating the likelihood of walking ability in IS patients. The development of this model will aid in screening IS patients at risk of walking impairment, thereby reducing the incidence of post-IS gait disorders through early, targeted interventions.

## 2 Methods

### 2.1 Study patients

**Figure 1:**
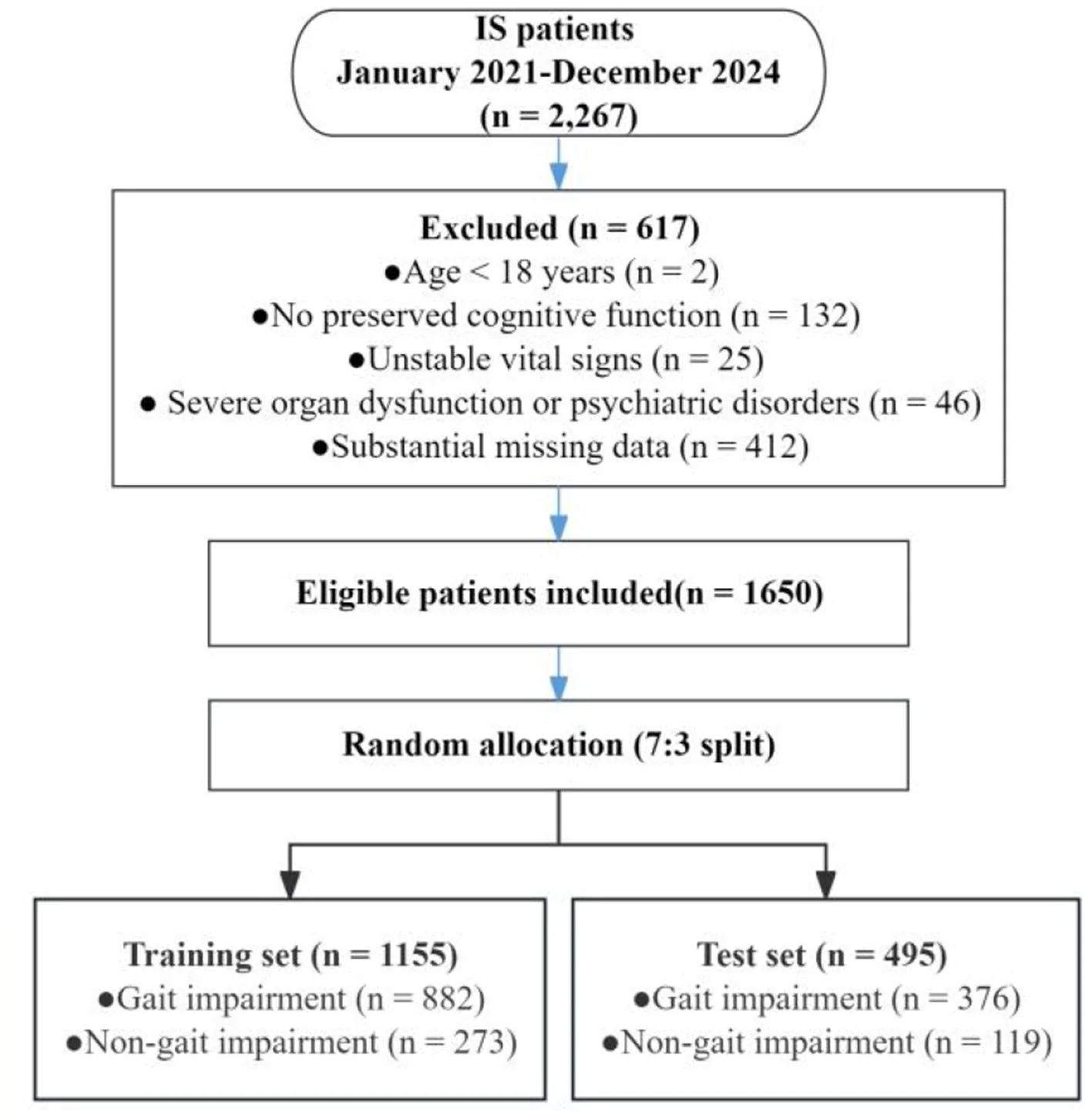
Participant flow diagram.

This study retrospectively collected data from IS patients at the Rehabilitation Department of Xiangyang Central Hospital between January 2021 and December 2024. The inclusion criteria were as follows: (1) hospitalization for IS during the study period; (2) age ≥18 years; and (3) preserved cognitive function enabling completion of study-related assessments. Patients were excluded if they met any of the following criteria: (1) unstable vital signs; (2) active infections, chronic autoimmune inflammatory diseases, hematological disorders, malignancies, or use of immunosuppressive medications; (3) comorbid severe organ dysfunction or psychiatric disorders; (4) substantial missing data related to the study in their medical records. A total of 1,650 participants were included in the analysis. The participant selection process is illustrated in the flow diagram (Figure 1).

Written informed consent was provided by all participants. The study protocol was approved by the Institutional Ethics Committee of Xiangyang Central Hospital (Ethics Approval No.: No. ChiCTR2024-093). The study was conducted in accordance with the principles of the Declaration of Helsinki.

### 2.2 Data collection

All data documented at the time of hospital admission were collected, encompassing the following categories: (1) Demographic characteristics: age, sex (male/female), smoking status, alcohol consumption, and prior medical history, including hypertension, diabetes mellitus, coronary artery disease (CHD), and hyperlipidemia; (2) Disease-specific characteristics: The cranial computed tomography (CT) data of all subjects were collected for lesion localization. Two experienced neuroradiologists independently reviewed the imaging and classified lesion locations according to a standardized anatomical brain template, with lesion locations categorized as cortical, subcortical, basal ganglia, brainstem, cerebellar, and thalamic regions. The imaging evaluators were blinded to the patients' data, performing lesion location assessment solely based on the imaging findings. Inter-rater reliability was assessed using Cohen's kappa coefficient. In our study, the Cohen's kappa for lesion location determination between the two evaluators was 0.68, indicating substantial agreement. Any disagreements between the two evaluators were resolved through consensus discussion with a third senior neuroradiologist before the final lesion location data were used for subsequent analyses. (3) Laboratory data: given the established association between post-stroke inflammatory status and functional recovery, complete blood count (CBC)-derived inflammatory biomarkers were analyzed (10). Blood samples for CBC measurements were obtained within 24–48 h after rehabilitation admission, following an overnight fast. The inflammatory biomarkers evaluated in this study included the neutrophil-to-lymphocyte ratio (NLR), monocyte-to-lymphocyte ratio (MLR), systemic immune-inflammation index (SII), and systemic inflammation response index (SIRI), all of which were obtained from standard CBC measurements. These indices were calculated as follows: NLR = absolute neutrophil count / absolute lymphocyte count; MLR = absolute monocyte count / absolute lymphocyte count; SII= (platelet count ^*^ neutrophil count) / lymphocyte count; SIRI = (neutrophil count ^*^ monocyte count) / lymphocyte count.

### 2.3 Walking ability assessment

Functional Ambulation Classification (FAC) was employed to assess patients‘ walking ability, serving as a reliable and validated ordinal scale for categorizing ambulatory function in post-stroke individuals (13). The instrument classifies walking ability into a 6-point scale, ranging from 0 (non-ambulatory, indicating an inability to walk or requiring substantial support from two persons) to 5 (fully independent ambulation, enabling the patient to walk freely on level and non-level surfaces, including stairs and slopes, without physical assistance) (13). FAC assessments were performed within 24 h after rehabilitation admission by trained rehabilitation therapists who were blinded to the patients' baseline clinical data.

The primary endpoint was defined as the presence of significant gait impairment, corresponding to an FAC score of ≤ 2. This cutoff was selected based on established clinical convention and consensus recommendations. FAC scores of 0–2 represent patients who require physical assistance to ambulate. Specifically, FAC 0 indicates non-functional ambulation, FAC 1 denotes dependence on continuous manual contact from one person, and FAC 2 indicates dependence on intermittent or continuous light touch to assist balance or coordination—whereas FAC ≥ 3 indicates supervision-only or independent walking (13). The third Stroke Recovery and Rehabilitation Roundtable (SRRR3) consensus recommended the Functional Ambulation Category (FAC, 0–5) for walking independence assessment and explicitly stated that “an FAC score of less than three should be used to determine the need for an additional standing test (FAC < 3, add BBS to Mini-BESTest) or the feasibility to assess walking (FAC < 3, 10 mWT, 6 MWT, and DGI are ‘not testable')” (14). Furthermore, the FAC has demonstrated excellent reliability, good concurrent and predictive validity, and good responsiveness in patients with hemiparesis after stroke (13). FAC ≤ 2 has also been widely used as a clinically meaningful cutoff in stroke rehabilitation research to define non-ambulatory or dependent ambulators (15, 16). Therefore, FAC ≤ 2 was chosen as a clinically meaningful threshold distinguishing patients with gait dependence requiring physical assistance from those with functional ambulation.

### 2.4 Feature selection

In this study, we employed a hybrid feature selection strategy integrating LASSO regression, the Boruta algorithm, and multivariate logistic regression to identify robust factors for estimating the risk of post-IS walking dysfunction. This sequential consensus approach (requiring a variable to pass through multiple independent feature selection filters) is a well-established methodological standard for reducing overfitting risk and ensuring that only the most robust factors are retained (17–19). LASSO regression was utilized for its capability to perform automated feature selection by applying an L1-penalty. This constraint forces the coefficients of less influential variables toward zero, effectively excluding them from the model. Boruta algorithm, a wrapper method built around a Random Forest classifier, was implemented. Boruta assesses feature importance by comparing the significance of original features with that of randomly permuted “shadow features.” Crucially, the final set of key factors was defined as the consensus intersection of features independently selected by all three algorithms, with the “AND” rule applied sequentially: only variables that passed both LASSO and Boruta were eligible for logistic regression. Among the four inflammatory biomarkers evaluated (NLR, MLR, SII, and SIRI), SII and SIRI were not retained by LASSO (their coefficients were shrunk to zero) and were therefore excluded from subsequent steps, regardless of their Boruta status. Only NLR and MLR satisfied both the LASSO and Boruta criteria and entered the logistic regression stage. This method is particularly effective at capturing non-linear relationships and interactions that might be overlooked by linear models. The final set of key factors was defined as the consensus intersection of features independently selected by all three algorithms. This stringent, tripartite strategy aims to ensure that the chosen variables are not only statistically significant but also clinically relevant and robust, providing a solid foundation for building a parsimonious and high-performing model for gait dysfunction after IS. All feature selection steps were performed solely within the training set. The test set was kept completely untouched and was not used for any feature selection or model tuning procedures.

### 2.5 Model development

Based on the feature selection results, machine learning algorithms suitable for assessing the risk of walking impairment in IS patients were identified and evaluated. The models assessed included eXtreme Gradient Boosting (XGBoost), Logistic Regression (LR), Random Forest (RF), Decision Tree (DT), Support Vector Machine (SVM), and k-Nearest Neighbors (KNN). The dataset of IS patients was randomly partitioned into a training set and a test set at a ratio of 7:3. The training set was utilized for model training and parameter optimization, while the test set was reserved for evaluating the model's generalization capability and discriminative performance.

Model performance was validated using multiple evaluation metrics, including accuracy, recall, precision, and the area under the Receiver Operating Characteristic curve (AUC). Given the class imbalance in the dataset (76.2% gait impairment), class weights (inverse frequency weighting) were applied during model training to penalize misclassification of the minority class more heavily. For all performance metrics, 95% confidence intervals (CIs) were calculated using 1,000 bootstrap resamples with replacement from the test set, providing percentile-based CIs to reflect the sampling variability of each metric (20, 21). A two-sided *P* value < 0.05 was considered statistically significant. Model calibration was evaluated using calibration plots and the Brier score. The Brier score ranges from 0 to 1, with lower values indicating better calibration. The performance of the different models was systematically compared to select the optimal algorithm as the final tool. In addition, decision curve (DCA) analysis was conducted to assess the net clinical benefit of the model at varying threshold probabilities.

### 2.6 Model interpretability

Model interpretability was achieved using SHapley Additive exPlanations (SHAP), a game-theoretic approach that quantifies the contribution of each feature to model predictions by computing SHAP values. This method enables the assessment of both global and local feature importance, captures feature interdependencies and interactions, and facilitates the visualization of individual predictions for specific instances.

To explore synergistic effects beyond main effects, we performed SHAP interaction analysis using the TreeExplainer implementation in the SHAP Python package (version 3.11.5). Interaction heatmaps were used to visualize pairwise interaction strengths, and SHAP dependence plots with interaction coloring were employed to interpret the direction and clinical relevance of key interactions.

### 2.7 Statistical analysis

Categorical variables are presented as frequencies (*n*) and percentages (%), while continuous variables are expressed as either mean ± standard deviation (SD) or median (range), as appropriate. Group comparisons for variable distributions were performed using the independent samples *t*-test, the Mann-Whitney *U* test, or chi-square analysis. A two-sided *P* value less than 0.05 was considered statistically significant. All analyses were conducted using R (version 4.5.1) and Python (version 3.11.5).

## 3 Results

### 3.1 Patient characteristics

The basic characteristics of the study participants are summarized in Table 1. A total of 1,650 subjects (1,129 males and 521 females) were ultimately enrolled in this study. The cohort was randomly divided into a training set (*N* = 1,155) and a test set (*N* = 495). The mean age of the study population was 63.83 ± 12.31 years. Among the 1,650 participants, 1,258 patients were identified with gait impairment. The walking dysfunction cases were 882 in the training set and 376 in the test set, with no significant differences in baseline characteristics between the two sets (*P* > 0.05), indicating a balanced split. Comparative analyses between the gait impairment and non-impairment groups revealed significant differences (*P* < 0.05) in the distribution of lesion locations (frontal lobe, occipital lobe, basal ganglia), as well as in sex, age, hypertension, and diabetes mellitus status (Supplementary Table 1). Furthermore, participants with gait impairment were significantly older, had lower DBP, and exhibited higher levels of systemic inflammatory biomarkers, including the NLR, MLR, SII, and SIRI, compared to those without gait impairment (*P* < 0.05, see in Supplementary Table 1).

**Table 1:** Baseline characteristics.

Variable	Total	Train	Test	*P*
*N*	1,650	1,155	495	-
Gait impairment*, n (%)*	1,258 (76.2)	882 (76.4)	376 (76.0)	0.860
Hemisphere_lesion, n (%)				0.155
None	420 (25.5)	301 (26.1)	119 (24.0)	
Left	517 (31.3)	347 (30.0)	170 (34.3)	
Right	493 (29.9)	359 (31.1)	134 (27.1)	
Bilateral	220 (13.3)	148 (12.8)	72 (14.5)	
Location of occlusion, n (%)				
Frontal lobe	448 (27.2)	326 (28.2)	122 (24.6)	0.134
Temporal lobe	419 (25.4)	308 (22.4)	111 (26.7)	0.070
Parietal lobe	365 (22.1)	264 (22.9)	101 (20.4)	0.271
Occipital lobe	199 (12.1)	141 (12.2)	58 (11.7)	0.779
Basal ganglia	639 (38.7)	447 (38.7)	192 (38.8)	0.974
Thalamus	84 (5.1)	59 (5.1)	25 (5.1)	0.961
Brainstem	290 (17.6)	204 (17.7)	86 (17.4)	0.888
Cerebellum	117 (7.1)	84 (7.3)	33 (6.7)	0.660
Demographics				
Males, *n* (**%**)	1,129 (68.4)	794 (68.7)	335 (67.7)	0.669
Age, years	63.83 ± 12.31	63.69 ± 12.26	64.17 ± 12.45	0.464
Smoking, *n* (**%**)	559 (33.9)	398 (34.5)	161 (32.5)	0.447
Drinking, *n* (**%**)	542 (32.8)	378 (32.7)	164 (33.1)	0.873
Hypertension, *n* (**%**)	1,270 (77.0)	887 (76.8)	383 (77.4)	0.799
Dyslipidemia, *n* (**%**)	110 (6.7)	70 (6.1)	40 (8.1)	0.132
Diabetes, *n* (**%**)	544 (33.0)	388 (33.6)	156 (31.5)	0.411
CHD, *n* (**%**)	268 (16.2)	189 (16.4)	79 (16.0)	0.838
SBP, mmHg	129.50 ± 14.10	129.57 ± 14.30	129.34 ± 13.63	0.769
DBP, mmHg	79.64 ± 10.06	79.72 ± 10.26	79.48 ± 9.57	0.659
CBC-derived indicators				
NLR	3.19 ± 2.59	3.18 ± 2.37	3.21 ± 3.03	0.822
MLR	0.38 ± 0.21	0.38 ± 0.19	0.37 ± 0.24	0.569
SII, 10^3^/μL	587.00 (382.72, 936.31)	589.97 (379.75, 937.58)	582.30 (386.94, 933.11)	0.767
SIRI, 10^3^/μL	1.21 (0.80, 2.01)	1.24 (0.82, 2.01)	1.16 (0.77, 2.04)	0.252

CHD, Coronary heart disease; SBP, Systolic blood pressure; DBP, Diastolic blood pressure; CBC, Complete blood count; NLR, Neutrophil-to-lymphocyte ratio; MLR, Monocyte-to-lymphocyte ratio; SII, Systemic immune-inflammation index; SIRI, Systemic inflammation response index.

### 3.2 Feature selection

**Figure 2:**
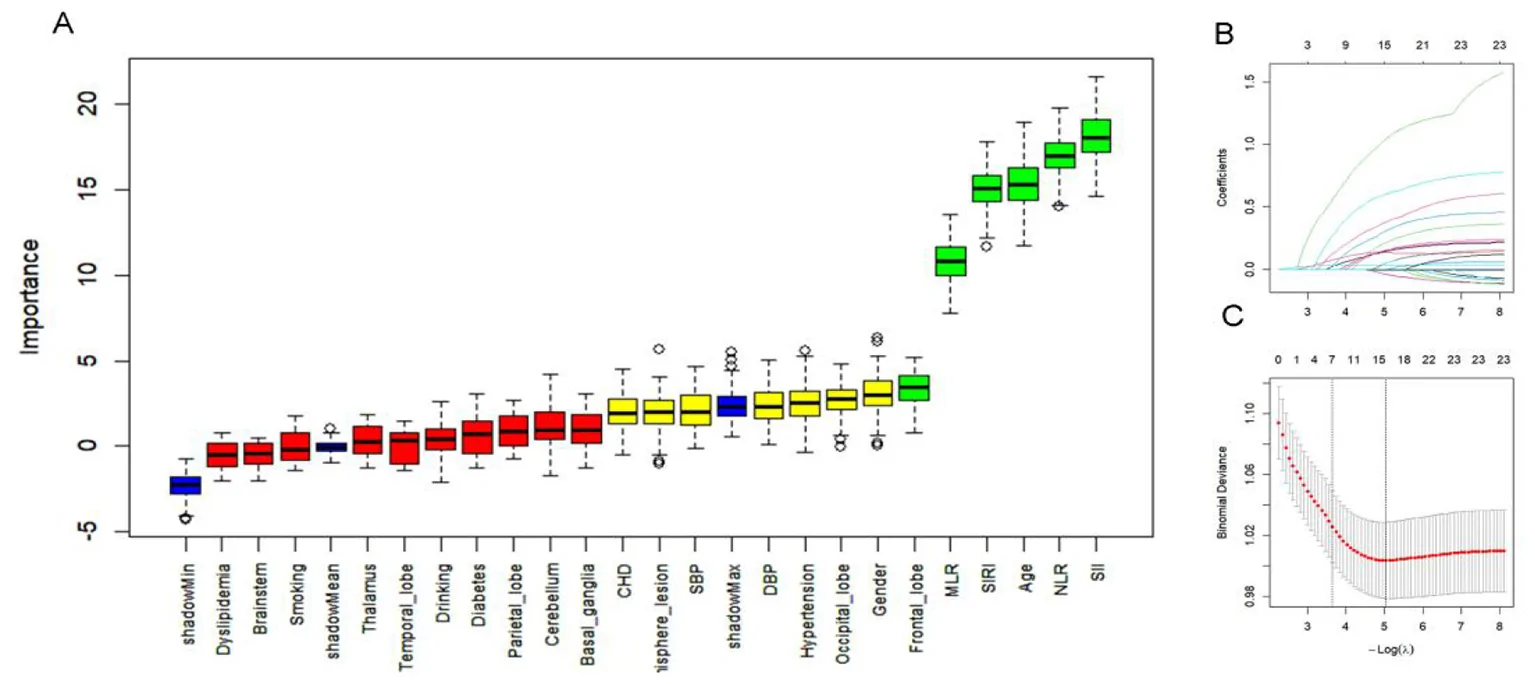
Feature selection using Boruta and LASSO regression. **(A)** The Boruta algorithm output: the blue boxplot depicts the shadow score with its minimum, mean, and maximum values. The green, yellow, and red boxplots represent the confirmed (important), tentative, and rejected features, respectively. **(B)** The coefficient regularization path of features in the LASSO regression model. **(C)** The process of determining the optimal parameter for the LASSO model via cross-validation. CHD, Coronary heart disease; DBP, Diastolic blood pressure; MLR, Monocyte-to-lymphocyte ratio; NLR, Neutrophil-to-lymphocyte ratio; SBP, Systolic blood pressure; SII, Systemic immune-inflammation index; SIRI, Systemic inflammatory response index.

Figure 2 illustrates the analytical results of the feature selection procedures employing the LASSO and Boruta algorithms. The LASSO regression analysis enabled the identification of 7 factors with the highest associative value from the initial set of candidate variables. Subsequently, the Boruta algorithm, a wrapper method based on random forests, confirmed the significance of six of these factors for assessing the risk of post-IS gait impairment by comparing the importance of original features with that of randomly permuted shadow features. These 6 variables were incorporated into a multivariate logistic regression model. The results demonstrated that frontal lobe lesion, occipital lobe lesion, age, sex, and NLR were all independent factors of walking dysfunction in IS patients (all *P* < 0.05) (Table 2).

**Table 2:** Multivariate logistic regression.

Variables	*OR* (95 *CI*)	*P*
Frontal lobe	1.527 (1.137, 2.052)	0.005
Occipital lobe	1.966 (1.261, 3.067)	0.003
Gender	1.389 (1.053, 1.834)	0.020
Age	1.041 (1.030, 1.052)	< 0.001
NLR	1.249 (1.098, 1.420)	< 0.001
MLR	2.923 (0.804, 10.624)	0.103

MLR, monocyte-to-lymphocyte ratio; NLR, neutrophil-to-lymphocyte ratio.

### 3.3 Evaluation of machine learning models

**Figure 3:**
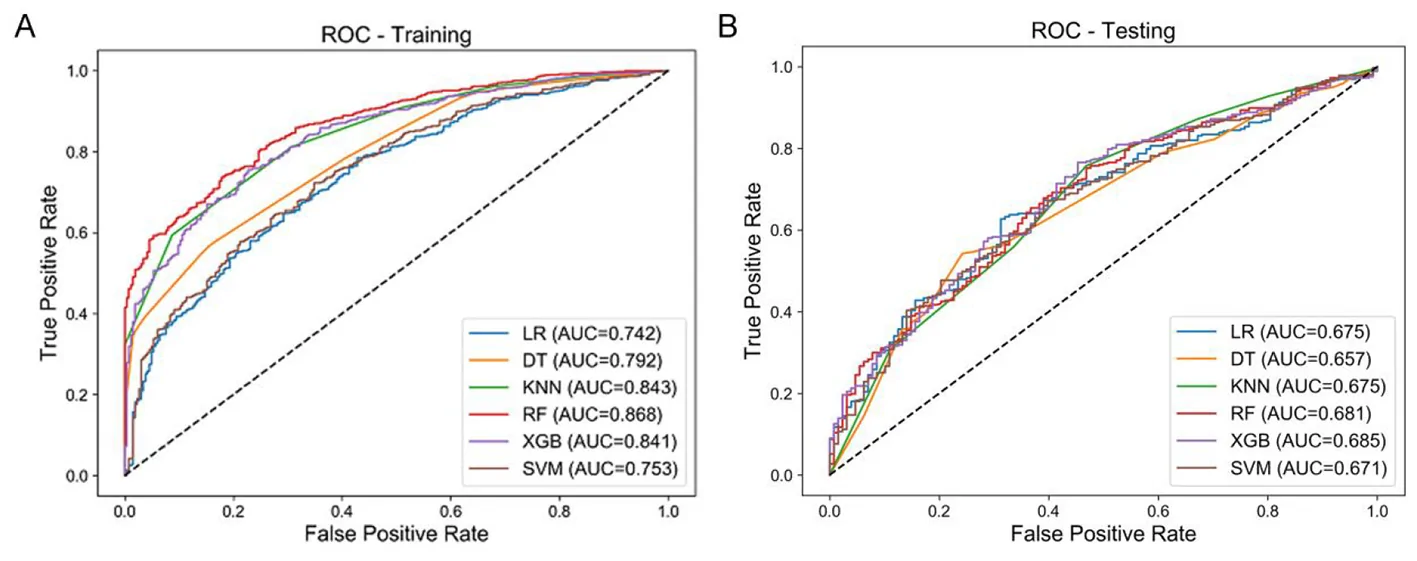
Comparison of receiver operating characteristic (ROC) curves for different ML models. **(A)** Training set. **(B)** Test set.

Five factors were selected as input variables to construct six distinct machine learning models for assessing the risk of gait impairment. The performance of these models on the training set was evaluated using metrics such as the AUC, accuracy, sensitivity (recall), and specificity. Figure 3, Table 3 and Supplementary Table 2 provide a detailed summary of performance metrics for all six models. Among the models investigated, RF achieved the highest AUC in the training set (0.868, 95% CI: 0.849–0.888), substantially outperforming other models. In the test set, XGBoost achieved the highest AUC (0.685, 95% CI: 0.635, 0.735), followed closely by RF (0.681, 95% CI: 0.630, 0.732). However, the 95% CIs for AUCs overlapped considerably across all models in the test set, indicating that no single model was statistically superior in the held-out test set. Considering the comprehensive performance across multiple metrics—including sensitivity (0.7575), specificity (0.5234), accuracy (0.6970), recall (0.7575), and F1-score (0.7875)—RF demonstrated more balanced discriminative ability than other models. Therefore, RF was cautiously selected as the preferred model, acknowledging that the choice was based on overall performance trade-offs rather than statistically significant superiority in the test set. The substantial performance decay from the training set to the test set (AUC drop from 0.868 to 0.681) suggests that the model is overfitted and requires further refinement and external validation before any clinical application. DCA curves for the test set and validation set in ML models were shown in Supplementary Figure 1.

**Table 3:** Discriminative performance of ML models in test set.

Model	AUC	Accuracy	Specificity	Sensitivity	Precision	Recall	F1-score	G-mean
LR	0.675 (0.625, 0.725)	0.6566 (0.6162, 0.6990)	0.5859 (0.4962, 0.6695)	0.6812 (0.6329, 0.7306)	0.8251 (0.7832, 0.8667)	0.6812 (0.6329, 0.7306)	0.7463 (0.7078, 0.7831)	0.6318 (0.5769, 0.6805)
XGB	0.685 (0.635, 0.735)	0.6788 (0.6404, 0.7212)	0.5781 (0.4855, 0.6596)	0.7139 (0.6684, 0.7596)	0.8291 (0.7870, 0.8667)	0.7139 (0.6684, 0.7596)	0.7672 (0.7320, 0.8012)	0.6424 (0.5864, 0.6909)
RF	0.681 (0.630, 0.732)	0.6970 (0.6586, 0.7394)	0.5234 (0.4394, 0.6063)	0.7575 (0.7154, 0.800)	0.8201 (0.7792, 0.8563)	0.7575 (0.7154, 0.800)	0.7875 (0.7542, 0.8217)	0.6297 (0.5765, 0.6835)
KNN	0.675 (0.620, 0.730)	0.7313 (0.6910, 0.7717)	0.3281 (0.2500, 0.4154)	0.8719 (0.8356, 0.9069)	0.7881 (0.7481, 0.8265)	0.8719 (0.8356, 0.9069)	0.8279 (0.7984, 0.8557)	0.5348 (0.4650, 0.6032)
SVM	0.671 (0.620, 0.722)	0.6545 (0.6141, 0.6950)	0.6016 (0.5156, 0.6866)	0.6730 (0.6246, 0.7213)	0.8289 (0.7886, 0.8689)	0.6730 (0.6246, 0.7213)	0.7429 (0.7042, 0.7796)	0.6362 (0.5874, 0.6837)
DT	0.657 (0.605, 0.709)	0.5960 (0.5535, 0.6404)	0.7344 (0.6616, 0.8046)	0.5477 (0.4986, 0.600)	0.8553 (0.8107, 0.8966)	0.5477 (0.4986, 0.600)	0.6678 (0.6252, 0.7106)	0.6344 (0.5916, 0.6768)

Calibration assessment was performed for all six models (Supplementary Figure 2; Supplementary Table 3). The RF model achieved a Brier score of 0.0197 in the training set and 0.1469 in the test set, indicating acceptable calibration performance. As shown in the calibration plots (Supplementary Figure 2), the RF model's calibration curve deviates modestly from the ideal diagonal line in the mid-to-high risk range, suggesting a tendency toward slight overconfidence in probability estimates. The other models showed similar or less favorable calibration patterns, with DT and KNN exhibiting more pronounced deviations from ideal calibration in the test set. These findings indicate that while the RF model demonstrates acceptable overall calibration, its probability estimates should be interpreted with some caution, particularly at higher risk levels.

The RF model achieved a balanced accuracy of 0.6405 (0.5972, 0.6887) and a PR-AUC of 0.8595 (0.8210, 0.8956) in the test set, indicating that the model maintains reasonable discriminative performance even after accounting for class imbalance (Supplementary Table 4).

Additional *post-hoc* analyses were performed on the training set to compare the discriminative performance of models built with each inflammatory marker individually (NLR-only, MLR-only, SII-only, SIRI-only) and in combination (Supplementary Table 5). The NLR-only model achieved the highest AUC among single-marker models (0.851, 95% CI: 0.812, 0.890), and adding other markers did not significantly improve performance (combined model AUC 0.856, 95% CI: 0.818, 0.894; *P* = 0.32 by DeLong test). These analyses were conducted on the training set to compare the relative value of different inflammatory markers and are not directly comparable to the main RF model test-set performance (AUC 0.681), which reflects generalization to held-out data.

### 3.4 Variable Importance of factors

**Figure 4:**
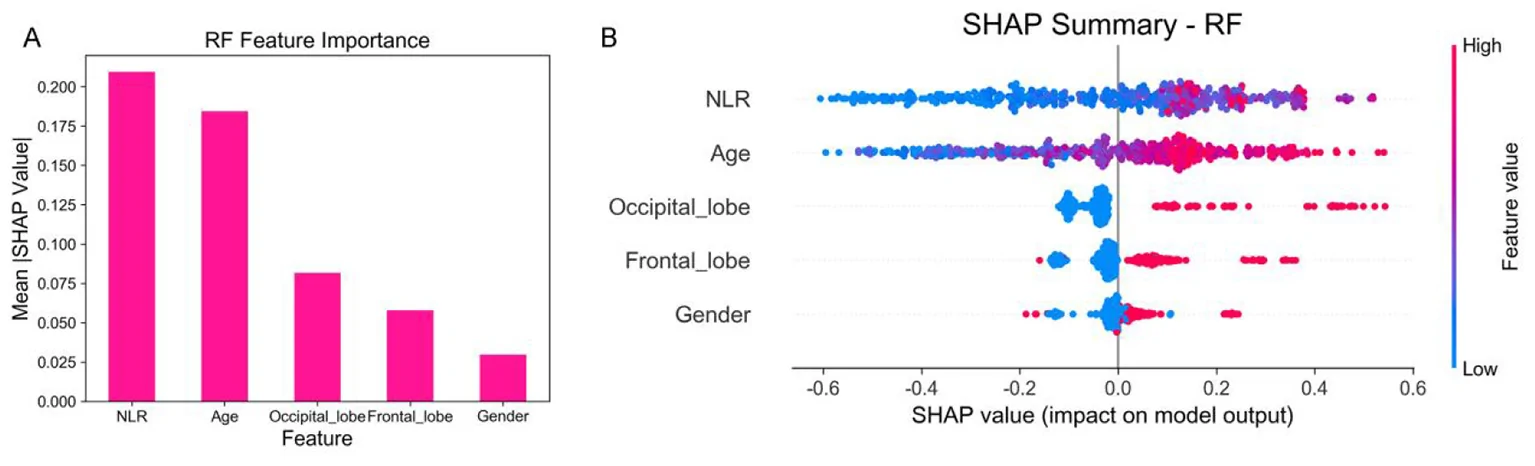
Evaluation of feature importance based on SHAP values and their contributions to the model output. **(A)** The bar plot displays the importance of the five features in descending order. **(B)** The SHAP distribution plot illustrates the contribution of each feature to the predictions. NLR, neutrophil-to-lymphocyte ratio.

A SHAP analysis was employed to quantify the contribution of each feature to the predictions of the RF model. The analysis identified age and the NLR as the two most critical factors influencing the model's prediction of gait impairment risk (Figure 4A). Furthermore, the model indicated that occipital lobe and frontal lobe lesions played moderate roles in risk prediction, whereas the influence of sex was less influential. Notably, older age, a higher NLR, and the presence of an occipital lobe lesion were associated with a higher probability of gait impairment (Figure 4B).

To further explore the synergistic effects between factors, we conducted SHAP interaction analysis examining pairwise feature interactions (Supplementary Figure 3). The interaction analysis between gender and NLR revealed a clinically meaningful pattern: the SHAP contribution of NLR to gait impairment risk assessment was substantially modulated by gender. Specifically, male patients with elevated NLR exhibited significantly higher SHAP values compared to female patients with comparable NLR levels, indicating that gender amplifies the predictive contribution of NLR in male patients. Preliminary analyses also suggest potential synergistic effects between age and NLR and between frontal and occipital lobe lesions (consistent with disruption of the fronto-occipital neural network, though these findings require further validation in independent cohorts.

## 4 Discussion

This study explored the factors influencing the development of gait impairment in patients with ischemic stroke. The analysis identified frontal lobe lesion, occipital lobe lesion, age, sex, and the NLR as significant influencing factors for gait impairment after ischemic stroke, with NLR and age being the two most critical factors affecting risk stratification. It is important to emphasize that SHAP analysis reveals patterns of association between features and model predictions rather than demonstrating causal relationships (22). SHAP values indicate the contribution of each variable to the model's decision-making process, not a direct causal effect on gait impairment. All interpretations of the SHAP results should be understood within this associative framework.

To our knowledge, this is the first study to systematically explore the relationship of CBC-derived inflammatory markers with post-IS gait impairment. Our findings demonstrate that NLR serves as a biomarker associated with walking impairment following ischemic stroke, which is consistent with previous research. Numerous studies have reported that NLR serves as an independent risk factor for acute ischemic stroke severity, mortality, and 3-month functional outcomes (23, 24). The underlying mechanism linking elevated NLR to post-stroke gait impairment likely involves multiple interconnected pathways. Neutrophils contribute to stroke morbidity by clogging capillaries and releasing pro-inflammatory cytokines, reactive oxygen species, and matrix metalloproteinases that disrupt the blood-brain barrier and promote secondary neuronal damage (25). Second, a higher NLR reflects a relative lymphopenia, which may indicate stroke-induced immunosuppression—a negative rehabilitative prognostic factor associated with poor motor performance and impaired gait stability at discharge from neurorehabilitation units (26). A recent meta-analysis further demonstrated that NLR demonstrates superior accuracy and a significant association with poor outcome, while SII shows greater stability across patient subgroups but weaker predictive strength (25). Third, systemic inflammation promotes atherosclerosis progression and thrombotic events, further compromising cerebral perfusion and functional recovery. These converging lines of evidence support the biological plausibility of NLR as a biomarker associated with post-stroke gait impairment. Further research is necessary to elucidate its direct relationship with specific gait parameters (e.g., gait speed, symmetry, dynamic stability). In clinical practice, active interventions targeting these inflammatory pathways may potentially alleviate gait dysfunction in patients with cerebral infarction.

Our study identified that advanced age is associated with an elevated risk of post-stroke gait disability and potentially poorer functional outcomes. Research has demonstrated age as one of the most influential factors of gait recovery following stroke (27). In elderly stroke patients, age-related neurological decline often exacerbates gait abnormalities, such as reduced walking speed and asymmetric movement patterns (28). Post-stroke motor impairments frequently lead to altered weight-bearing capacity on the affected side. With aging, the compensatory adjustments required to adapt to these changes, such as modifying motor planning and walking posture, become increasingly difficult to execute (29). Furthermore, aging is not only linked to declines in muscle mass and function but is also closely associated with a heightened systemic inflammatory levels (12, 30, 31). Studies have identified the NLR as an independent predictor of functional outcomes in elderly patients with acute ischemic stroke (24, 32). This suggests that age and NLR may interact in the pathogenesis of walking impairment. Therefore, in clinical practice, greater attention to inflammatory markers such as NLR in older patients may help improve prognostic accuracy and guide targeted rehabilitation strategies to optimize functional recovery.

This study identified both occipital and frontal lobe lesions as influencing factors for gait impairment following IS. Normal walking is a complex functional motor behavior requiring advanced cognitive regulation, and its successful execution is critically dependent on the integrity of the fronto-occipital visuomotor network (33). The occipital visual association cortex serves as the central processor for dynamic environmental visual information (including spatial orientation, surface texture, obstacle recognition, and motion depth perception), and continuously delivers visual feedback signals essential for real-time gait correction and postural adjustment (34, 35). This visual information is transmitted anteriorly via the inferior fronto-occipital fasciculus (IFOF, a core long-range white matter tract) to the premotor cortex, supplementary motor area, and dorsolateral prefrontal cortex (36, 37). The frontal lobe integrates these visual inputs to construct gait programs, regulate step rhythm, support prospective balance prediction, and mediate attentional allocation and conflict inhibition during dual-task walking (33, 38). Disruption at any node of this fronto-occipital visuomotor network (whether at the cortical level or along the IFOF), leads to impaired static and dynamic gait stability, reduced obstacle avoidance capacity, and ultimately decreased functional ambulation. Our findings are consistent with functional neuroimaging evidence showing that increased functional connectivity between the ipsilateral occipital cortex and contralateral primary somatosensory and motor cortices supports gait recovery during walking (39). Reduced prefrontal cortex volume correlates significantly with decreased walking speed and increased gait variability post-stroke (40), and prefrontal hyperactivation during dual-task walking often exacerbates gait deterioration (41, 42). Our results showed that occipital and frontal lobe infarctions increased gait impairment risk, which provides clinical outcome-level validation of the critical role of the fronto-occipital visuomotor network in post-stroke gait maintenance. Based on the pathophysiological mechanism by which fronto-occipital visuomotor network damage mediates gait impairment, targeted neuromodulation may serve as a precise rehabilitation intervention. However, these suggestions are speculative and hypothesis-generating, as our study did not directly test any neuromodulation intervention. Future clinical trials are needed to evaluate whether rTMS or tDCS targeting the occipital cortex or prefrontal cortex can improve gait outcomes in stroke patients with fronto-occipital lesion.

We also found that gender as a potential factor for walking impairment following IS. Specifically, male patients demonstrated a higher susceptibility to post-stroke gait dysfunction compared to females, a finding consistent with the well-established epidemiological pattern that males exhibit a greater overall risk of experiencing ischemic stroke than females (43). The SHAP interaction analysis further revealed a clinically important synergistic effect between gender and NLR. Male patients with elevated NLR demonstrated a substantially higher risk of gait impairment than female patients with comparable NLR levels. This gender-NLR interaction may be explained by sex-specific differences in immune responses to ischemic injury. Male sex hormones, particularly testosterone, may exacerbate post-ischemic neuroinflammation by promoting neutrophil infiltration and proinflammatory cytokine release, while female sex hormones, particularly estrogen, may exert neuroprotective and anti-inflammatory effects (44). These findings are consistent with epidemiological evidence that males exhibit a higher incidence of ischemic stroke compared to females (43). Additionally, preliminary analyses indicate potential synergistic effects between age and NLR, and such an effect may also exist between frontal lobe and occipital lobe lesions. These interaction patterns warrant further validation in independent cohorts.

In this study, we employed and compared multiple machine learning models to assess the risk of gait impairment in IS patients. Performance comparison based on discriminative accuracy revealed that the RF model achieved the highest discriminative ability, with an AUC of 0.868, which was notably superior to that of traditional LR (AUC = 0.742). This outcome underscores the capability of RF to capture complex, non-linear variable interactions, a finding consistent with the report by Ma et al., and highlights the potential of the RF model in guiding individualized rehabilitation strategies and optimizing the allocation of stroke rehabilitation resources (27). The notably lower AUC of LR compared to tree-based and other machine learning algorithms (XGBoost, RF, DT) and kernel-based methods (SVM, KNN) suggests limited efficacy of conventional LR in handling non-linear relationships frequently present in stroke rehabilitation data. This supports the notion that machine learning approaches may offer a distinct advantage over traditional regression in modeling multifaceted clinical outcomes. While machine learning has progressively transitioned from theoretical exploration to clinical validation in the field of stroke prognosis, its widespread implementation still faces challenges. However, the substantial drop in AUC from the training set (0.868) to the internal test set (0.681) warrants careful consideration. This discrepancy may reflect overfitting due to the relatively high feature-to-sample ratio, the single-center nature of the data, and the absence of key covariates such as infarct volume, NIHSS score at admission, and rehabilitation intensity. Ma and Xie also reported similar findings in acute anterior circulation ischemic stroke, where machine learning models also showed performance decay from training to validation, highlighting the need for cautious interpretation (27). Therefore, while the RF model demonstrates promising discriminative ability within our cohort, its generalizability should be interpreted with caution, and the model should be considered a preliminary tool requiring further refinement and external validation.

This study developed and compared multiple machine learning-based risk stratification models for post-IS gait impairment. By identifying significant factors across various dimensions (including lesion location, functional status, medical history, and laboratory biomarkers), these models can assist healthcare institutions in optimizing long-term care resource planning, enable early screening of high-risk populations for targeted secondary prevention, and help patients and their families make informed psychological preparations and actively participate in treatment decision-making.

Some limitations of the present study must be considered when interpreting the results. First, its single-center design may restrict the generalizability of the findings. Multi-center studies are needed to validate the external applicability of our results. Second, as a retrospective study, the history of conditions such as hypertension and diabetes was based on patient recall, which may introduce recall bias. Third, although frontal and occipital lobe lesions were identified as influencing factors, the underlying mechanisms remain unclear. Future investigations should focus on the substantive characteristics of lesions in these brain regions, such as the presence of white matter damage, to further elucidate the potential pathways involved. Fourth, several key clinical variables were not available in our retrospective dataset, including stroke severity as measured by the NIHSS score at admission, infarct volume, time from stroke onset to rehabilitation initiation and rehabilitation intensity. These variables are likely strong determinants of walking ability, and their omission may have substantially limited the model's predictive performance and clinical utility. Future prospective studies with systematic collection of these covariates are essential for developing more robust models. Furthermore, this retrospective study was limited by available imaging data. Patients underwent only cranial CT without DTI, precluding quantitative assessment of IFOF white matter tract damage. Additionally, we did not perform subgroup analyses based on lesion volume or white matter involvement. Studies incorporating multi-modal imaging (e.g., DTI, resting-state fMRI) are needed to conduct comprehensive imaging subgroup analyses and to further elucidate the role of white matter pathway disruption in post-stroke gait recovery.

## 5 Conclusion

A comparative analysis of risk stratification models for walking impairment in ischemic stroke patients demonstrated that RF showed the balanced performance across multiple metrics within this single-center cohort. The model identified frontal lobe lesions, occipital lobe lesions, age, sex, and NLR as significant determinants of post-stroke walking impairment. This model, based on readily accessible variables, may serve as a preliminary research tool for risk stratification for walking impairment after ischemic stroke, pending further refinement and external validation.

## Data Availability

The original contributions presented in the study are included in the article/Supplementary material, further inquiries can be directed to the corresponding authors.
