# Correction: Development and validation of multiple machine learning models for identifying factors associated with walking ability in ischemic stroke patients: a single-center retrospective study with SHAP approach

**DOI:** 10.3389/fneur.2026.2002605

**Published:** 2026-09-29

**Authors:** Jianying Qiu, Xiaoguang Yang, Meng Guo, Wei Guo, Xinyi Guo, Xiping Jia, Qianqian Sun

**Affiliations:** 1 School of Electrical Engineering, Guangdong Songshan Polytechnic College, Shaoguan, Guangdong, China; 2 Department of Rehabilitation Medicine, Xiangyang Central Hospital, Affiliated Hospital of Hubei University of Arts and Science, Xiangyang, Hubei, China; 3 Department of Medical Records and Statistics, Xiangyang Central Hospital, Affiliated Hospital of Hubei University of Arts and Science, Xiangyang, Hubei, China; 4 School of Computer Science, Guangdong Polytechnic Normal University, Guangzhou, China; 5 Rehabilitation Medicine and Rehabilitation Engineering Technology Xiangyang Key Laboratory, Xiangyang, Hubei, China

**Keywords:** influencing factor, ischemic stroke, machine learning, neutrophil-to-lymphocyte ratio, walking ability

In the published article, the affiliation “School of Computer Science, Guangdong Polytechnic Normal University, Guangzhou, China” was omitted. This affiliation has now been added to author Xiping Jia.

The affiliation “Department of Medical Records and Statistics, Xiangyang Central Hospital, Affiliated Hospital of Hubei University of Arts and Science, Xiangyang, Hubei, China” was erroneously assigned to author Xiping Jia. It has now been removed.

The original article has been updated.

