# Supplementary material for: Development and validation of multiple machine learning models for identifying factors associated with walking ability in ischemic stroke patients: a single-center retrospective study with SHAP approach
Source: Front Neurol. 2026 Sep 1;17:1854073. doi: 10.3389/fneur.2026.1854073 (PMC13574706; doi:10.3389/fneur.2026.1854073)
Supplement: Supplementary file 1 [file Supplementary_file_1.docx]

**Supplementary Table 1. Basic characteristics of the patients in the gait impairment group and the non-gait impairmen group**

| **Variable** | **Non-Gait impairment** | **Gait impairment** | ***P*** |
| --- | --- | --- | --- |
| *N* | 392 | 1258 | - |
| ***Hemisphere_lesion, n (%)*** |  |  | 0.172 |
| No | 112 (28.6) | 308 (24.5) |  |
| Left | 129 (32.9) | 388 (30.8) |  |
| Right | 105 (26.8) | 388 (30.8) |  |
| Bilateral | 46 (11.7) | 174 (13.8) |  |
| ***Location of occlusion, n (%)*** |  |  |  |
| Frontal lobe | 78 (19.9) | 370 (29.4) | <0.001 |
| Temporal lobe | 85 (21.7) | 334 (26.6) | 0.053 |
| Parietal lobe | 73 (18.6) | 292 (23.2) | 0.056 |
| Occipital lobe | 27 (6.9) | 172 (13.7) | <0.001 |
| Basal ganglia | 169 (43.1) | 470 (37.4) | 0.041 |
| Thalamus | 21 (5.4) | 63 (5.0) | 0.784 |
| Brainstem | 66 (16.8) | 224 (17.8) | 0.660 |
| Cerebellum | 21 (5.4) | 96 (7.6) | 0.126 |
| ***Demographics*** |  |  |  |
| Males, n (**%**) | 97 (24.7) | 424 (33.7) | 0.001 |
| Age, years | 58.13 ± 12.56 | 65.61 ± 11.68 | <0.001 |
| Smoking, n (**%**) | 143 (36.5) | 416 (33.1) | 0.213 |
| Drinking, n (**%**) | 140 (35.7) | 402 (32.01) | 0.166 |
| Hypertension, n (**%**) | 275 (70.2) | 995 (79.1) | <0.001 |
| Dyslipidemia, n (**%**) | 25 (6.4) | 85 (6.8) | 0.793 |
| Diabetes, n (**%**) | 103 (26.3) | 441 (35.1) | 0.001 |
| CHD, n (**%**) | 52 (13.3) | 216 (17.2) | 0.067 |
| SBP | 129.00 ± 14.46 | 129.65 ± 13.99 | 0.432 |
| DBP | 80.69 ± 10.30 | 79.32 ± 9.96 | 0.020 |
| ***CBC-derived indicators*** |  |  |  |
| NLR | 2.41 ± 2.09 | 3.44 ± 2.67 | <0.001 |
| MLR | 0.31 ± 0.14 | 0.40 ± 0.22 | <0.001 |
| SII, 10^3^/μL | 448.43 (308.83, 645.41) | 655.84 (419.07, 1064.03) | <0.001 |
| SIRI, 10^3^/μL | 0.95 (0.69, 1.38) | 1.33 (0.87, 2.27) | <0.001 |

CHD: Coronary heart disease; SBP: Systolic blood pressure; DBP: Diastolic blood pressure; CBC: Complete blood count; NLR: Neutrophil-to-lymphocyte ratio; MLR: Monocyte-to-lymphocyte ratio; SII: Systemic immune-inflammation index; SIRI: Systemic inflammation response index.

**Supplementary Table 2. Discriminative Performance of ML Models in Train Set**

| **Model** | **AUC** | **Accuracy** | **Specificity** | **Sensitivity** | **Precision** | **Recall** | **F1-score** | **G-mean** |
| --- | --- | --- | --- | --- | --- | --- | --- | --- |
| LR | 0.742 (0.717, 0.767) | 0.6649  (0.6381,0.6935) | 0.6780  (0.6198 ,0.7379) | 0.6611  (0.6301 ,0.6913) | 0.8739  (0.8486 ,0.8999) | 0.6611  (0.6301 ,0.6913) | 0.7527  (0.7292 ,0.7769) | 0.6695  (0.6378 ,0.7021) |
| XGB | 0.792 (0.769, 0.815) | 0.7394  (0.7143 ,0.7645) | 0.7841  (0.7333 ,0.8359) | 0.7262  (0.6950 ,0.7551) | 0.9190  (0.8989 ,0.9391) | 0.7262  (0.6950 ,0.7551) | 0.8113  (0.7896 ,0.8322) | 0.7546  (0.7249 ,0.7824) |
| RF | 0.843 (0.822, 0.864) | 0.7697  (0.7463 ,0.7948) | 0.7614  (0.7120 ,0.8175) | 0.7722  (0.7446 ,0.7996) | 0.9161  (0.8968 ,0.9367) | 0.7722  (0.7446 ,0.7996) | 0.8380  (0.8188 ,0.8571) | 0.7667  (0.7369 ,0.7990) |
| KNN | 0.868 (0.849, 0.888) | 0.8130  (0.7913 ,0.8355) | 0.4924  (0.4339 ,0.5486) | 0.9080  (0.8888 ,0.9261) | 0.8579  (0.8360 ,0.8788) | 0.9080  (0.8888 ,0.9261) | 0.8822  (0.8671 ,0.8971) | 0.6687  (0.6279 ,0.7077) |
| SVM | 0.841 (0.820, 0.862) | 0.6641  (0.6381 ,0.6909) | 0.6894  (0.6295 ,0.7500) | 0.6566  (0.6256 ,0.6884) | 0.8771  (0.8514 ,0.9015) | 0.6566  (0.6256 ,0.6884) | 0.7510  (0.7270 ,0.7748) | 0.6728  (0.6399 ,0.7045) |
| DT | 0.753 (0.728, 0.778) | 0.6303  (0.6017 ,0.6580) | 0.8447  (0.8015, 0.8889) | 0.5668  (0.5328 ,0.5987) | 0.9249  (0.9026 ,0.9456) | 0.5668  (0.5328 ,0.5987) | 0.7029  (0.6742 ,0.7284) | 0.6919  (0.6640 ,0.7174) |

**Supplementary Figure 1. DCA curves for the test set and validation set in ML models**

**Supplementary Table 3. Brier Score of all Models**

| **Model** | **Brier_Train** | **Brier_Test** |
| --- | --- | --- |
| LR | 0.2177 | 0.2223 |
| DT | 0.1251 | 0.2522 |
| KNN | 0.1154 | 0.1551 |
| RF | 0.0197 | 0.1469 |
| XGB | 0.1729 | 0.2074 |
| SVM | 0.1304 | 0.1380 |

**Supplementary Figure 2. Calibration plots for the test set and validation set in ML models**

**Supplementary Table 4. Balanced accuracy and PR-AUC of ML Models in Test Set**

| **Model** | balanced accuracy  (95% CI) | PR-AUC  (95% CI) |
| --- | --- | --- |
| LR | 0.6336 (0.5836,0.6808) | 0.8600 (0.8262,0.8938) |
| XGB | 0.6460 (0.5971,0.6916) | 0.8642 (0.8278,0.8967) |
| RF | 0.6405 (0.5972,0.6887) | 0.8595 (0.8210,0.8956) |
| KNN | 0.6000 (0.5573,0.6472) | 0.8313 (0.7892,0.8670) |
| SVM | 0.6373 (0.5920,0.6839) | 0.8534 (0.8153,0.8909) |
| DT | 0.6410 (0.5991,0.6843) | 0.8295 (0.7882,0.8697) |

**Supplementary Table 5. Comparison of the efficacy of individual and combined modeling of various inflammatory markers (post-hoc analyses on the training set). These analyses were performed on the training set to compare the relative predictive value of different inflammatory markers and are not directly comparable to the main model test-set performance.**

| Model | AUC (95% CI) | Accuracy (95% CI) | Sensitivity (95% CI) | Specificity (95% CI) |
| --- | --- | --- | --- | --- |
| NLR-only | 0.851  (0.812, 0.890) | 0.742  (0.704, 0.780) | 0.768  (0.722, 0.814) | 0.656  (0.598, 0.714) |
| MLR-only | 0.723  (0.676, 0.770) | 0.658  (0.618, 0.698) | 0.682  (0.632, 0.732) | 0.586  (0.526, 0.646) |
| SII-only | 0.697  (0.648, 0.746) | 0.642  (0.602, 0.682) | 0.665  (0.615, 0.715) | 0.578  (0.518, 0.638) |
| SIRI-only | 0.684  (0.634, 0.734) | 0.636  (0.596, 0.676) | 0.658  (0.608, 0.708) | 0.570  (0.510, 0.630) |
| Combined (NLR+MLR+SII+SIRI) | 0.856  (0.818, 0.894) | 0.748  (0.710, 0.786) | 0.772  (0.726, 0.818) | 0.664  (0.606, 0.722) |

**Supplementary Figure 3. Interactive SHAP visual chart depicting the gender-NLR interaction effect. The figure displays the SHAP values of NLR (y-axis) against NLR values (x-axis), with points colored by gender (blue: female; orange: male).**
